# Preliminary Insights of Brazilian Snake Venom Metalloproteomics

**DOI:** 10.3390/toxins15110648

**Published:** 2023-11-10

**Authors:** Bruna Cavecci-Mendonça, Karen Monique Luciano, Tauane Vaccas, Laudicéia Alves de Oliveira, Eloisa Fornaro Clemente, Bruno Cesar Rossini, José Cavalcante Souza Vieira, Luciana Curtolo de Barros, Ilka Biondi, Pedro de Magalhães Padilha, Lucilene Delazari dos Santos

**Affiliations:** 1 Biotechnology Institute (IBTEC), São Paulo State University (UNESP), Botucatu 18607-440, SP, Brazil; brucavecci@gmail.com (B.C.-M.); bruno.rossini@unesp.br (B.C.R.); 2 Graduate Program in Tropical Diseases, Botucatu Medical School (FMB), São Paulo State University (UNESP), Botucatu 18618-687, SP, Brazil; tauanevaccas@hotmail.com (T.V.); laudiceia.biomed@gmail.com (L.A.d.O.); 3 Triad for Life Ltda, Prospecta–Botucatu Technological Incubator, Botucatu 18610-034, SP, Brazil; 4 Center of Studies of Venoms and Animals Venomous (CEVAP), São Paulo State University (UNESP), Botucatu 18619-002, SP, Brazil; karen.m.luciano@gmail.com (K.M.L.); luciana.barros@unesp.br (L.C.d.B.); 5 Graduate Program in Research and Development (Medical Biotechnology), Botucatu Medical School (FMB), São Paulo State University (UNESP), Botucatu 18618-687, SP, Brazil; eloisa.fornaro@unesp.br; 6 Department of Chemical and Biological Sciences, Institute of Biosciences (IBB), São Paulo State University (UNESP), Botucatu 18618-689, SP, Brazil; cavalcante.vieira@unesp.br (J.C.S.V.); pedro.padilha@unesp.br (P.d.M.P.); 7 Laboratory of Venomous Animals and Herpetology, State University of Feira de Santana (UEFS), Feira de Santana 44036-900, BA, Brazil; ibiondi@uefs.br

**Keywords:** metalloproteomics, venomics, snakebites, diagnostic devices, shotgun proteomics, mass spectrometry, atomic spectrometry

## Abstract

Snakebite envenoming is one of the most significantly neglected tropical diseases in the world. The lack of diagnosis/prognosis methods for snakebite is one of our motivations to develop innovative technological solutions for Brazilian health. The objective of this work was to evaluate the protein and metallic ion composition of *Crotalus durissus terrificus*, *Bothrops jararaca*, *B. alternatus*, *B. jararacussu*, *B. moojeni*, *B. pauloensis,* and *Lachesis muta muta* snake venoms. Brazilian snake venoms were subjected to the shotgun proteomic approach using mass spectrometry, and metal ion analysis was performed by atomic spectrometry. Shotgun proteomics has shown three abundant toxin classes (PLA_2_, serine proteases, and metalloproteinases) in all snake venoms, and metallic ions analysis has evidenced that the Cu^2+^ ion is present exclusively in the *L. m. muta* venom; Ca^2+^ and Mg^2+^ ions have shown a statistical difference between the species of *Bothrops* and *Crotalus* genus, whereas the Zn^2+^ ion presented a statistical difference among all species studied in this work. In addition, Mg^2+^ ions have shown 42 times more in the *C. d. terrificus* venom when compared to the average concentration in the other genera. Though metal ions are a minor fraction of snake venoms, several venom toxins depend on them. We believe that these non-protein fractions are capable of assisting in the development of unprecedented diagnostic devices for Brazilian snakebites.

## 1. Introduction

Snakebite envenoming is a neglected tropical disease that kills >100,000 people and causes sequelae in >400,000 people every year. Sub-Saharan Africa, Southeast Asia, Latin America, and some regions of Oceania form part of the socio-economically deprived population with the most pronounced cases of snakebite envenoming. Many families and rural workers are hit the hardest by snakebite, and consequently, they may not be able to return to work after accidents, becoming yet more vulnerable to poverty [1].

According to the epidemiological data shown in the Disease Information and Notification System (SINANWEB) of the Brazilian Ministry of Health, 2,970,443 accidents involving snakes in humans occurred in the Brazilian territory from 2007 to 2022 [2]. It is known that among these reported accidents, more than 900 Brazilians have sequelae, and 278 die on average every year. The perplexity becomes greater when the numbers indicated as ignored and/or blank notifications are observed: 2,565,641 notifications in which health agents did not register in the patients’ records and/or were unable to identify the type of snake that caused the accident. These data represent about 86.37% of the total notifications of snakebites in Brazil in which the snake causing the accident has not been identified [2].

Many reasons justify the delay in applying the correct antivenom or the use of non-specific antivenoms on the snake victims. These factors directly influence the severity of the accident, which can cause sequelae or even lead to death, such as the victim’s difficulty in identifying the snake, the late manifestation of the symptoms, the similarity of the clinical manifestations among snakes of different genera, and the lack of experience of health agents in snakebites [3].

Scarce, non-specific, inaccessible, non-neutralizing, or non-existent antivenoms in many Brazilian regions and a lack of diagnosis/prognosis methods about snakebite aggravation are the motivations of our research team, which has endeavored to develop innovative technological solutions for Brazilian health. The high variability in the composition of snake venoms is responsible for the various clinical manifestations of envenomation, ranging from local tissue damage to potentially lethal systemic effects [1,4,5,6,7,8]. Convinced that understanding a problem is the key to solving it, the objective of this work was to evaluate the protein and metallic ion composition of *Crotalus durissus terrificus*, *Bothrops jararaca*, *B. alternatus*, *B. jararacussu*, *B. moojeni*, *B. pauloensis,* and *Lachesis muta muta* snake venoms using the metalloproteomics approach. Our efforts are in accordance with the Strategic Plan to Combat Ophidism postulated by the World Health Organization (WHO) [3] to find potential molecules present in the Brazilian snake venoms capable of acting as biomarkers of the severity of ophidian accidents or being molds embedded in rapid diagnosis devices for snake identification.

## 2. Results

**Figure 1:**
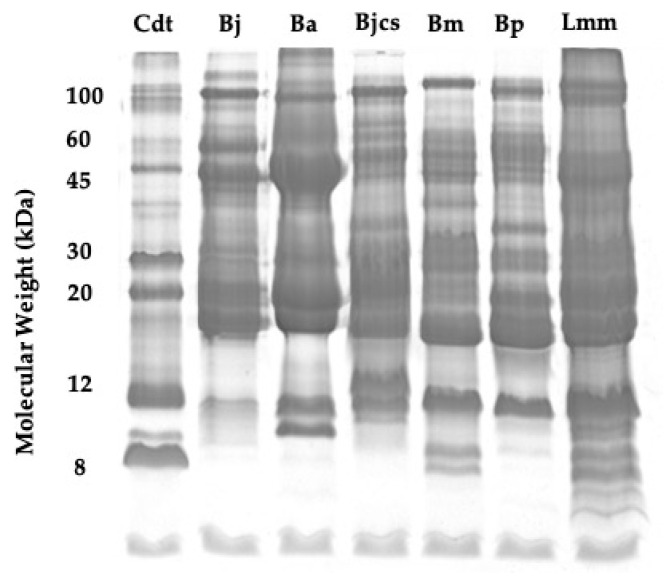
Snake venoms’ protein profile using electrophoresis gel at 15% (*m*/*v*) SDS-PAGE.; Cdt = *C. d. terrificu*s venom; Bj = *B. jararaca* venom; Ba = *B. alternatus* venom; Bjcs = *B. jararacussu* venom; Bm = *B. moojeni* venom; Bp = *B. pauloensis* venom; Lmm = *L. m. muta* venom.

*C. d. terrificus*, *B. jararaca*, *B. alternatus*, *B. jararacussu*, *B. moojeni*, *B. pauloensis,* and *L. m. muta* snake venom protein profiles are shown in Figure 1. The protein yield of each venom, the percentage of the major toxins, and the concentration of metallic ions are shown in Table 1.

In Figure 1, it is possible to visualize the major diversity of protein bands in the Brazilian snake venoms, with molecular masses ranging from 100 to 8 kDa. Furthermore, we noticed a high similarity between the protein profiles of the venoms of species of the genus *Bothrops* and the venom of *L. m. muta*. The similarity is even more reinforced near the molecular masses of 12, 15, and 45 kDa of the three genera studied. These facts confirm the importance of finding differences in non-protein molecules to differentiate snakebites.

Shotgun proteomics have shown that several toxin classes have a potential relationship with metallic ions in the Brazilian snake venoms studied. The three most abundant toxins were PLA_2_, serine proteases, and metalloproteinases in the species of *Bothrops* genus, adding up to more than 70% of these venoms. For *C. d. terrificus* and *L. m. muta* venom, these three toxins added up to 50.67% and 37.09% of the total toxins/proteins, respectively. Other less abundant toxins were evidenced in these snake venoms, such as nucleotidases, phosphodiesterases, and phosphatases (Supplementary Materials, Figures S1–S7 and Tables S1–S7). As regards metallic ions, the Cu^2+^ ion was detected exclusively in the *L. m. muta* venom, and Ca^2+^ and Mg^2+^ ions showed a statistical difference between the species of *Bothrops* and *Crotalus* genera (*p* < 0.01), whereas the Zn^2+^ ion presented a statistical difference among all species studied in this work (*p* < 0.01). In addition, Mg^2+^ ions showed 42 times more in the *C. d. terrificus* venom when compared to the average concentration in the other genera.

## 3. Discussion

The use of analytical techniques of high sensitivity and resolution, such as mass spectrometry, in the qualification and quantification of the snake venom’s proteome has brought precious contributions to the development of products and processes based on their toxins [9]. Related areas in the field of proteomics, such as phosphoproteomics, metallomics, and any biological process in general where a heteroatom (that is, any atom other than C, H, O, and N) is an integral part of its mechanism, have joined the investigative strategies for human and animal health [10,11,12,13,14,15]. In this way, metalloproteomics has arisen from the need to explore the protein content, and more precisely, the metalloproteinases and the metals non-covalently linked to them [16], and to expand knowledge of how metal functions in biology and medicine [17].

According to our findings and many studies in the literature, Brazilian snake venoms have several structurally and biologically common toxins [18,19,20,21,22,23]. Until this moment, there have been no reports of the existence of exclusive toxin structures in each Brazilian venom that does not present cross-reactivity to non-specific antivenoms. Early diagnosis is of major importance in the practice of immediate administration of the specific antivenoms, and it can be enhanced with better knowledge of the composition of snake venoms to license the development of low-cost and rapid-diagnosis devices [3]. Nowadays, the only product commercially available is the Snake Venom Detection kit licensed by the company Seqirus, which is based on an antibody-antigen strategy using the differences in Australian snakes’ protein composition [24,25]. However, recent advancements in synergetic interaction among biotechnology and microelectronics have advocated biosensor technology for a wide array of applications such as environmental pollution, cancer detection, food monitoring, pathogen detection, sensing, metal ion detection, etc. [26,27]. Therefore, we believe metallic ions present in Brazilian snake venoms can be potential targets of diagnostic devices for snakebites in the future.

Based on the findings of Lemon and collaborators [28], our group identified several toxins present in Brazilian snake venoms in different amounts, and they can carry metal ions in their structures and/or depend on these ions to perform their functions. It is known that several snake venom enzymes are particularly dependent on Ca^2+^, Mg^2+^, and Zn^2+^ metal ions [29] and particularly dependent on divalent metal ions, including Ca^2+^-dependent phospholipase A2, divalent metal ion-dependent 5’-nucleotidase, Mg^2+^-dependent phosphodiesterase, Mg^2+^- or Ca^2+^-dependent alkaline phosphatase, and Zn^2+^-dependent proteinase [28,30,31]. Some metalloproteinases that present Zn^2+^ ions in their structures are responsible for proteolytic degradation of the extracellular matrix, inflammations, hemorrhages, and other physiological responses [32,33,34]. Therefore, our metalloproteomics data corroborate the literature for presenting the highest levels of Ca^2+^ and Zn^2+^ ions and metalloproteinases in the *Bothrops* and *L. m. muta* venoms in relation to *C.d. terrificus* venom.

Regarding the Mg^2+^ ions, our data corroborate the studies of Lemon and collaborators [28], which showed that the greater the toxicity of the snake venom, the greater the Mg^2+^ ion content. According to the LD50 data, *C. d. terrificus* snake venoms have greater toxicity than *Bothrops* and *Lachesis* genera venoms [35]. Scientific evidence has shown that Cu^2+^ and Mg^2+^ are metal ion cofactors for snake venom enzymes, as some hemorrhage-inducing toxins and Mg^2+^ ions can restore some enzymatic activity of snake venom components when other metal ions are removed [36]. Interestingly, some venom snake proteinase activity is enhanced by Ca^2+^ and Mg^2+^ ions but completely inhibited by Zn^2+^ ions and by metal chelators [28].

## 4. Conclusions

This is the first brief report about Brazilian snake venom metalloproteomics in the literature. We consider that, with better in-depth knowledge of the metal ion cofactor content of Brazilian snake venoms in the future, combined with the structural composition of toxins/enzymes from these venoms, we can assist in the development of unprecedented diagnostic devices for Brazilian snakebites. Though metal ions are present in a minor fraction of snake venoms, several venom toxins depend on them. We believe that this non-protein fraction is capable of distinguishing the types of snakebite, even though it is present in the biological fluids (urine and blood serum) of victims of snakebites.

## 5. Materials and Methods

*C. d. terrificus*, *B. jararaca*, *B. moojeni*, *B. jararacussu*, *B. pauloensis.* and *B. alternatus* venom pools were obtained from healthy adult specimens from scientific breeding of CEVAP/UNESP, SP, Brazil, and *L. m. muta* venom pool was obtained from healthy adult specimens from scientific breeding of UEFS, BA, Brazil. Both scientific breeding programs are under authorization from the Brazilian Institute for the Environment and Renewable Natural Resources, and the significant findings of this study are part of a larger study over multiple years, registered at the National System of Genetic Heritage and Associated Traditional Knowledge (SISGEN-AFB4085 and AC1CCFC).

The handling of snakes and the extraction of venoms occurred according to Santos and collaborators [37], and blood biochemical medium parameters from snakes from this study can be accessed in Supplementary Materials Table S8. Protein quantification was established using the Bradford method [38], and electrophoresis assays were performed in denatured and reduced conditions [39]. For the shotgun proteomics analysis, the proteins were subjected to in-solution trypsin digestion using 1:50 enzyme/substrate in the presence of Rapigest^®^ SF 0.2% (*w*/*v*) (Waters, Milford, MA, USA) [5]. Mass spectrometry analyses from each venom pool were performed in technical triplicate using Xevo Q-TOF G2 (Waters) and the parameters used are described in Braga and collaborators [40]. The data were processed using ProteinLynx GlobalServer software (Waters), using the *Serpentes* from the NCBI database (Taxonomy ID 8570 with 551,225 sequences) with parameters: trypsin enzyme, carbamidomethylation as fixed modification, methionine oxidation as variable modification, a cleavage missed by the enzyme, monoisotopic mass, {M+H}^+1^, peptide tolerance error (MS) ± 0.1 Da and tolerance error (MS/MS) ± 0.1 Da, and instrument type ESI-Q-TOF. The percentage of proteins was calculated using the average spectra count of each class of protein in the samples. For metallic ions quantification (Ca^2+^, Cu^2+^, Fe^2+^, Mg^2+^ and Zn^2+^), the procedures were carried out by Cavecci-Mendonça and collaborators [41] by using a Shimadzu AA-6800 atomic absorption spectrometer (Shimadzu). Analysis of Variance (ANOVA) followed by Tukey test (*p* < 0.01) was performed to evaluate whether the amount of metal ions in venoms presented statistical differences between the genera of Brazilian snakes.

## Figures and Tables

**Table 1:** Proteomics and Metallomics of Brazilian snake venoms.

Species	Total Protein Yield (%)	Metallo Proteinases (%)	PLA_2_s (%)	Serine Proteases (%)	Ca^2+^ *	Cu^2+^ *	Fe^2+^ *	Mg^2+^ *	Zn^2+^ *
*C. d. terrificus*	95.35	0.81	40.67	39.19	0.506 ± 0.00	N/D	0.069 ± 0.15	2.484 ± 0.02	0.384 ± 0.02
*B. jararaca*	70.56	19.78	4.88	45.00	0.683 ± 0.13	N/D	N/D	0.060 ± 0.04	0.922 ± 0.04
*B. alternatus*	74.45	11.52	29.88	35.13	0.776 ± 0.07	N/D	0.139 ± 0.79	0.099 ± 0.08	1.016 ± 0.04
*B. jararacussu*	92.35	7.27	35.26	38.63	0.689 ± 0.11	N/D	0.157 ± 0.22	0.098 ± 0.04	0.728 ± 0.31
*B. moojeni*	78.71	21.88	39.29	13.39	1.109 ± 0.27	N/D	0.385 ± 1.34	0.060 ± 0.03	1.167 ± 0.08
*B. pauloensis*	89.01	13.43	28.06	28.86	0.800 ± 0.33	N/D	0.159 ± 0.08	0.031 ± 0.002	0.947 ± 0.22
*L. m. muta*	84.85	24.73	7.87	4.49	N/D	0.543 ± 0.01	N/D	N/D	0.653 ± 0.03

* mg/L; N/D: not detected.

## Supplementary Materials

The following supporting information can be downloaded at https://www.mdpi.com/article/10.3390/toxins15110648/s1, Figures S1–S7: Proteome of Brazilian snake venoms; Tables S1–S7: Shotgun Proteomics Analysis; Table S8: Blood Biochemical Parameters from healthy adult snakes specimen donors of the studied venoms.

## References

[1] Gutiérrez J.M., Calvete J.J., Habib A.G., Harrison R.A., Williams D.J., Warrell D.A. (2017). Snakebite envenoming. Nat. Rev. Dis. Primers.

[2] SINAN (2023). Sistema de Informação de Agravos de Notificação. http://portalsinan.saude.gov.br/dados-epidemiologicos-sinan.

[3] WHO (World Health Organization) (2019). Snakebite Envenoming: A Strategy for Prevention and Control.

[4] Saravia P., Rojas E., Arce V., Guevara C., López J.C., Chaves E., Velásquez R., Rojas G., Gutiérrez J.M. (2002). Geographic and ontogenic variability in the venom of the neotropical rattlesnake *Crotalus durissus*: Pathophysiological and therapeutic implications. Rev. Biol. Trop..

[5] Larréché S., Chippaux J.P., Chevillard L., Mathé S., Résière D., Siguret V., Mégarbane B. (2021). Bleeding and thrombosis: Insights into pathophysiology of *Bothrops* venom-related hemostasis disorders. Int. J. Mol. Sci..

[6] Cavalcante J.D.S., de Almeida C.A.S., Clasen M.A., da Silva E.L., de Barros L.C., Marinho A.D., Rossini B.C., Marino C.L., Carvalho P.C., Jorge R.J.B. (2022). A fingerprint of plasma proteome alteration after local tissue damage induced by *Bothrops leucurus* snake venom in mice. J. Proteom..

[7] Cavalcante J.S., Brito I.M.D.C., De Oliveira L.A., De Barros L.C., Almeida C., Rossini B.C., Sousa D.L., Alves R.S., Jorge R.J.B., Santos L.D.D. (2022). Experimental *Bothrops atrox* Envenomation: Blood Plasma Proteome Effects after Local Tissue Damage and Perspectives on Thromboinflammation. Toxins.

[8] Seifert S.A., Armitage J.O., Sanchez E.E. (2022). Snake envenomation. N. Engl. J. Med..

[9] Calvete J.J., Lomonte B., Saviola A.J., Calderón Celis F., Ruiz Encinar J. (2023). Quantification of snake venom proteomes by mass spectrometry: Considerations and perspectives. Mass Spectrom. Rev..

[10] Lovell M.A., Robertson J.D., Teesdale W.J., Campbell J.L., Markesbery W.R. (1998). Copper, iron and zinc in Alzheimer’s disease senile plaques. J. Neurol. Sci..

[11] Fu D., Finney L. (2014). Metalloproteomics: Challenges and prospective for clinical research applications. Expert Rev. Proteom..

[12] James S.A., Churches Q.I., de Jonge M.D., Birchall I.E., Streltsov V., McColl G., Adlard P.A., Hare D.J. (2017). Iron, Copper, and Zinc Concentration in Aβ Plaques in the APP/PS1 Mouse Model of Alzheimer’s Disease Correlates with Metal Levels in the Surrounding Neuropil. ACS Chem. Neurosci..

[13] Cavecci-Mendonça B., Vieira J.C.S., Lima P.M., Leite A.L., Buzalaf M.A.R., Zara L.F., Padilha P.M. (2020). Study of proteins with mercury in fish from the Amazon region. Food Chem..

[14] Steel T.R., Hartinger C.G. (2020). Metalloproteomics for molecular target identification of protein-binding anticancer metallodrugs. Metallomics.

[15] Mahan B., Chung R.S., Pountney D.L., Moynier F., Turner S. (2020). Isotope metallomics approaches for medical research. Cell. Mol. Life Sci..

[16] Wang Y., Li H., Sun H. (2019). Metalloproteomics for Unveiling the Mechanism of Action of Metallodrugs. Inorg. Chem..

[17] Zhou Y., Li H., Sun H. (2022). Metalloproteomics for Biomedical Research: Methodology and Applications. Annu. Rev. Biochem..

[18] Queiroz G.P., Pessoa L.A., Portaro F.C., Furtado M.F., Tambourgi D.V. (2008). Interspecific variation in venom composition and toxicity of Brazilian snakes from *Bothrops* genus. Toxicon.

[19] de Oliveira I.S., Cardoso I.A., Bordon K.D.C.F., Carone S.E.I., Boldrini-França J., Pucca M.B., Zoccal K.F., Faccioli L.H., Sampaio S.V., Rosa J.C. (2019). Global proteomic and functional analysis of *Crotalus durissus collilineatus* individual venom variation and its impact on envenoming. J. Proteom..

[20] Del-Rei T.H.M., Sousa L.F., Rocha M.M.T., Freitas-de-Sousa L.A., Travaglia-Cardoso S.R., Grego K., Sant’Anna S.S., Chalkidis H.M., Moura-da-Silva A.M. (2019). Functional variability of *Bothrops atrox* venoms from three distinct areas across the Brazilian Amazon and consequences for human envenomings. Toxicon.

[21] Farias I.B., Morais-Zani K., Serino-Silva C., Sant’Anna S.S., Rocha M.M.T.D., Grego K.F., Andrade-Silva D., Serrano S.M.T., Tanaka-Azevedo A.M. (2018). Functional and proteomic comparison of *Bothrops jararaca* venom from captive specimens and the Brazilian Bothropic Reference Venom. J. Proteom..

[22] Galizio N.D.C., Serino-Silva C., Stuginski D.R., Abreu P.A.E., Sant’Anna S.S., Grego K.F., Tashima A.K., Tanaka-Azevedo A.M., Morais-Zani K. (2018). Compositional and functional investigation of individual and pooled venoms from long-term captive and recently wild-caught *Bothrops jararaca* snakes. J. Proteom..

[23] Tasima L.J., Hatakeyama D.M., Serino-Silva C., Rodrigues C.F.B., de Lima E.O.V., Sant’Anna S.S., Grego K.F., de Morais-Zani K., Sanz L., Calvete J.J. (2020). Comparative proteomic profiling and functional characterization of venom pooled from captive *Crotalus durissus terrificus* specimens and the Brazilian crotalic reference venom. Toxicon.

[24] Steuten J., Winkel K., Carroll T., Williamson N.A., Ignjatovic V., Fung K., Purcell A.W., Fry B.G. (2007). The molecular basis of cross-reactivity in the Australian Snake Venom Detection Kit (SVDK). Toxicon.

[25] Nimorakiotakis V.B., Winkel K.D. (2016). Prospective assessment of the false positive rate of the Australian snake venom detection kit in healthy human samples. Toxicon.

[26] Khan S., Burciu B., Filipe C.D.M., Li Y., Dellinger K., Didar T.F. (2021). DNAzyme-Based Biosensors: Immobilization Strategies, Applications, and Future Prospective. ACS Nano.

[27] Liu C., Yu H., Zhang B., Liu S., Liu C.G., Li F., Song H. (2022). Engineering whole-cell microbial biosensors: Design principles and applications in monitoring and treatment of heavy metals and organic pollutants. Biotechnol. Adv..

[28] Lemon D.J., Horvath F.P., Ford A.A., May H.C., Moffett S.X., Olivera D.S., Hwang Y.Y. (2020). ICP-MS characterization of seven North American snake venoms. Toxicon.

[29] Bieber A.L., Lee C.Y. (1979). Metal and nonprotein constituents in snake venoms. Handbook of Experimental Pharmacology.

[30] Xu X., Liu Q., Xie Y. (2002). Metal ion-induced stabilization and refolding of anticoagulation factor II from the venom of Agkistrodon acutus. Biochemistry.

[31] Francis B., Seebart C., Kaiser I.I. (1992). Citrate is an endogenous inhibitor of snake venom enzymes by metal-ion chelation. Toxicon.

[32] Teixeira C.F., Fernandes C.M., Zuliani J.P., Zamuner S.F. (2005). Inflammatory effects of snake venom metalloproteinases. Mem. Inst. Ozwaldo Cruz.

[33] Gutiérrez J.M., Rucavado A., Escalante T., Díaz C. (2005). Hemorrhage induced by snake venom metalloproteinases: Biochemical and biophysical mechanisms involved in microvessel damage. Toxicon.

[34] Silva-Neto A.V., Santos W.G., Botelho A.F., Diamantino G.M., Soto-Blanco B., Melo M.M. (2018). Use of EDTA in the treatment of local tissue damage caused by the *Bothrops alternatus* venom. Arq. Bras. Med. Vet. Zootec..

[35] Chemical Toxicity Database (2023). Chemical Toxicity Calculator. https://www.drugfuture.com/toxic.

[36] Atanasov V.N., Stoykova S., Kolev H., Mitewa M., Petrova S., Pantcheva I.N. (2013). Effect of some divalent metal ions on enzymatic activity of secreted phospholipase A2 (sPLA2) isolated from Bulgarian *Vipera Ammodytes Meridionalis*. Biotechnol. Biotechnol. Equip..

[37] Santos L., Oliveira C., Vasconcelos B.M., Vilela D., Melo L., Ambrósio L., da Silva A., Murback L., Kurissio J., Cavalcante J. (2021). Good management practices of venomous snakes in captivity to produce biological venom-based medicines: Achieving replicability and contributing to pharmaceutical industry. J. Toxicol. Environ. Health B Crit. Rev..

[38] Bradford M. (1976). A rapid and sensitive method for the quantitation of microgram quantities of protein utilizing the principle of protein-dye binding. Anal. Biochem..

[39] Laemmli U.K. (1970). Cleavage of structural proteins during the assembly of the head of bacteriophage T4. Nature.

[40] Braga C.P., Bittarello A.C., Padilha C.C., Leite A.L., Moraes P.M., Buzalaf M.A., Zara L.F., Padilha P.M. (2015). Mercury fractionation in dourada (*Brachyplatystoma rousseauxii*) of the Madeira River in Brazil using metalloproteomic strategies. Talanta.

[41] Cavecci B., Lima P.M., Vieira J.C.S., Braga C.P., Queiroz J.V., Bittarello A.C., Padilha P.M. (2015). Use of ultrasonic extraction in determining apparent digestibility in fish feed. J. Food Meas. Charact..

