# Supplementary material for: Preliminary Insights of Brazilian Snake Venom Metalloproteomics
Source: Toxins (Basel). 2023 Nov 10;15(11):648. doi: 10.3390/toxins15110648 (PMC10675094; doi:10.3390/toxins15110648)
Supplement: Supplementary file 1 [file toxins-15-00648-s001.zip › Supplementary Material Figure S1 to S7_Proteome of brazilian snake venoms.pdf]

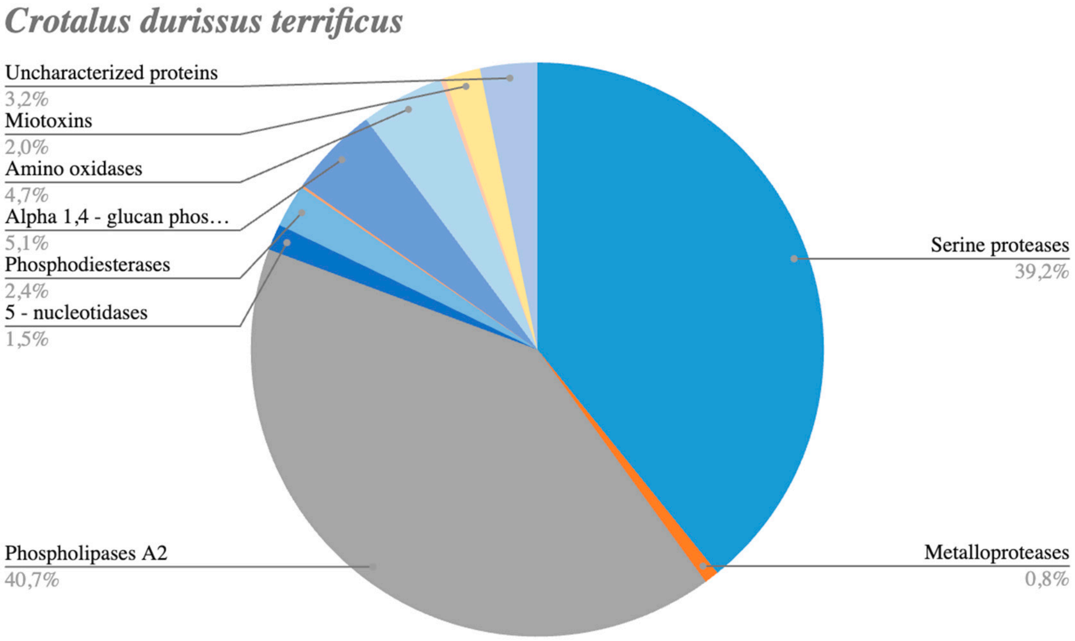

Figure S1: Proteome of *Crotalus durissus terrificus* venom.

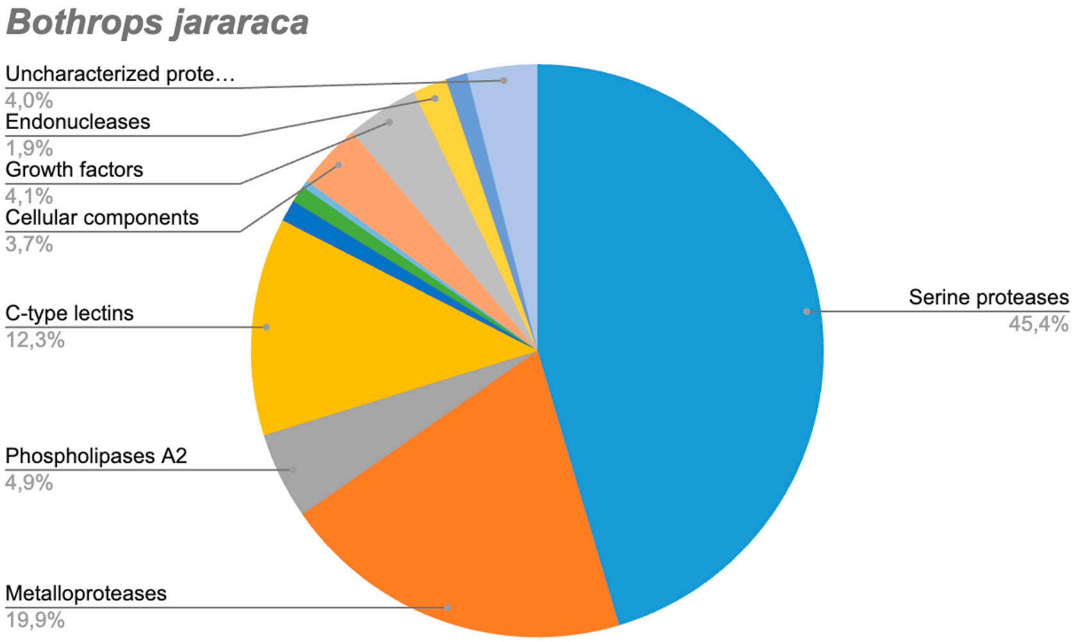

Figure S2: Proteome of *Bothrops jararaca* venom.

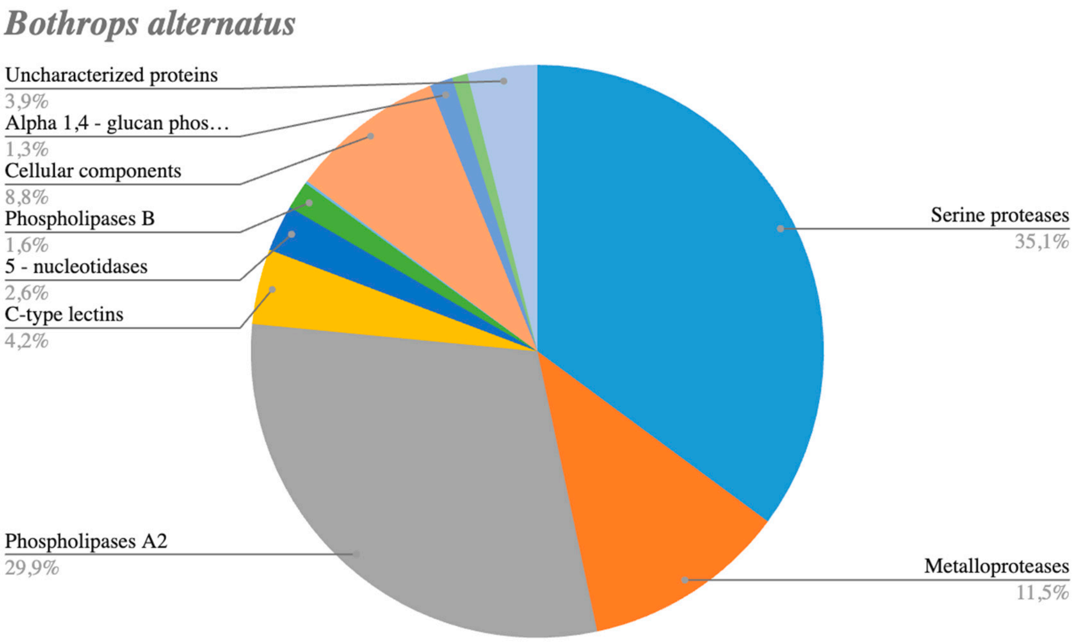

Figure S3: Proteome of *Bothrops alternatus* venom.

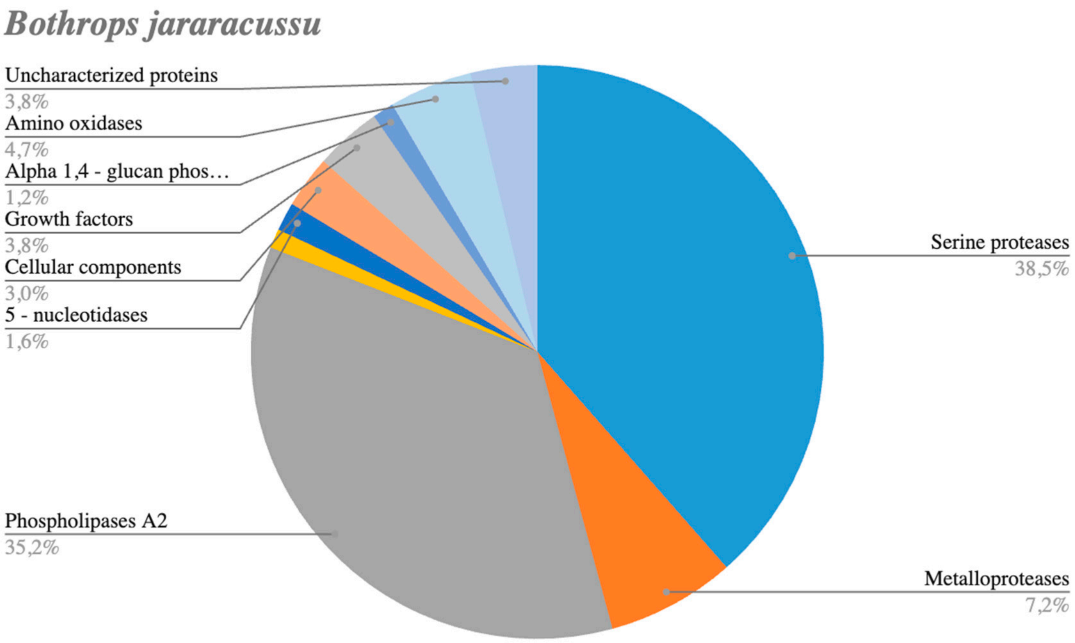

Figure S4: Proteome of *Crotalus durissus terrificus* venom.

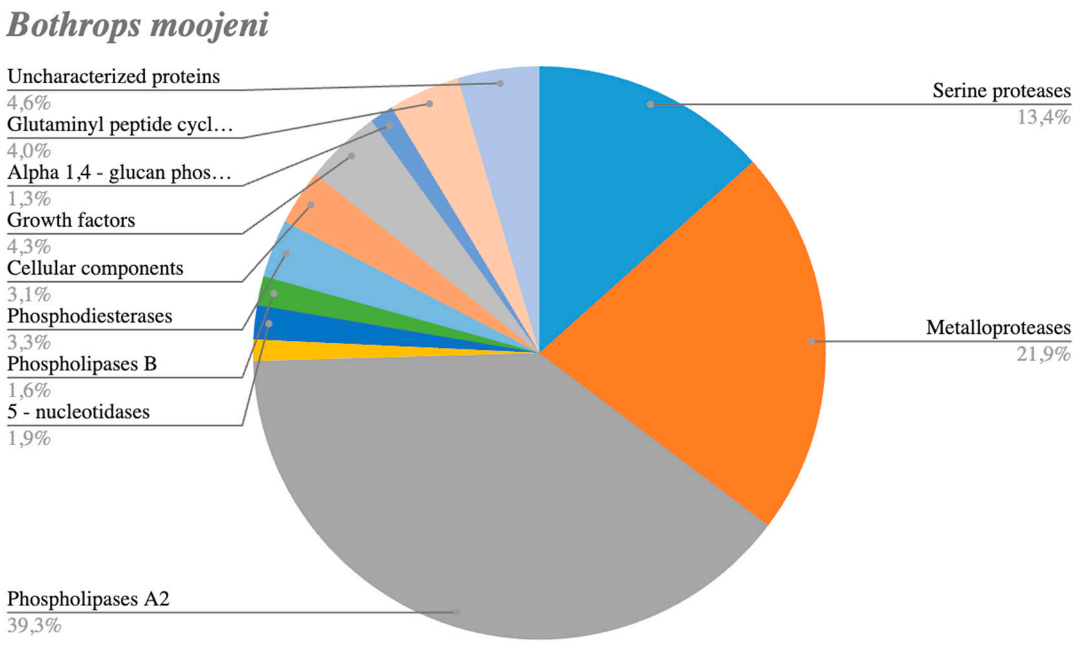

Figure S5: Proteome of *Bothrops moojeni* venom.

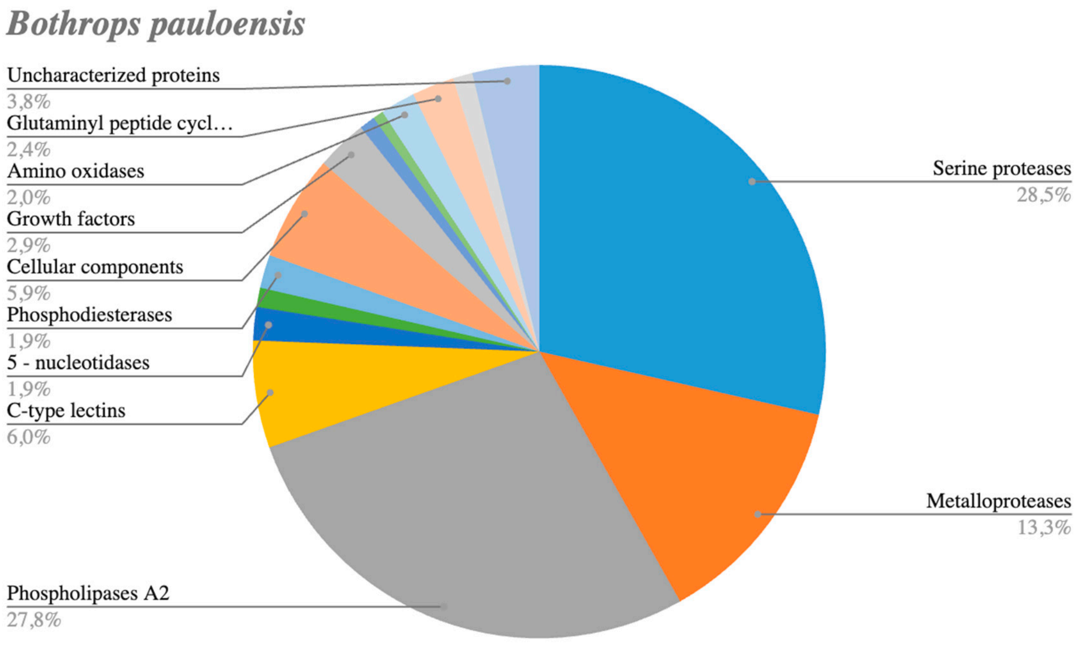

Figure S6: Proteome of *Bothrops pauloensis* venom.

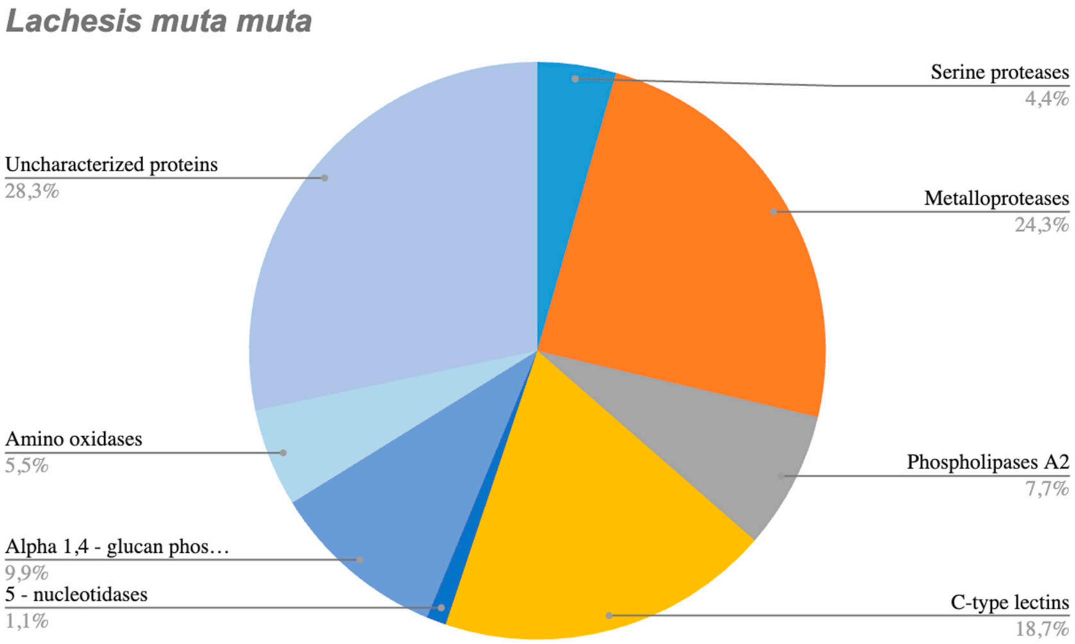

Figure S7: Proteome of *Lachesis muta muta* venom.
