# Supplementary material for: Preliminary Insights of Brazilian Snake Venom Metalloproteomics
Source: Toxins (Basel). 2023 Nov 10;15(11):648. doi: 10.3390/toxins15110648 (PMC10675094; doi:10.3390/toxins15110648)
Supplement: Supplementary file 1 [file toxins-15-00648-s001.zip › Supplementary Material Table S8_Blood Biochemical Parameters from healthy adult snakes specimen donors of the studied venoms.pdf]

Table S8. Blood Biochemical Parameters from healthy adult snake specimens.

	**Mean and Standard deviation**
**Albumin (g/dL)**	1.72 ± 0.37
**Total Protein (g/dL)**	3.90 ± 1.35
**AST/GOT (U/L)**	14.08 ± 15.80
**Uric acid (mg/dL)**	2.84 ± 3.21
